# Memory impairment caused by cerebral hematoma in the left medial temporal lobe due to ruptured posterior cerebral artery aneurysm

**DOI:** 10.1186/1471-2377-14-44

**Published:** 2014-03-07

**Authors:** Shinichiro Maeshima, Aiko Osawa, Fumitaka Yamane, Hidetoshi Shimaguchi, Ikuo Ochiai, Tomoyuki Yoshihara, Nahoko Uemiya, Ryuzaburo Kanazawa, Shoichiro Ishihara

**Affiliations:** 1 Department of Rehabilitation Medicine, Saitama Medical University International Medical Center, Hidaka, Saitama, Japan; 2 Department of Endovascular Neurosurgery, Saitama Medical University International Medical Center, Hidaka, Saitama, Japan; 3 Present address: Department of Rehabilitation Medicine II, School of Medicine, Fujita Health University, 424-1 Oodori-cho, Tsu 514-1298, Japan

**Keywords:** Amnesia, Subarachnoid hemorrhage, Ruptured cerebral aneurysm

## Abstract

**Background:**

Cognitive disorders, such as memory disturbances, are often observed following a subarachnoid hemorrhage. We present a very rare case where rupture of a posterior cerebral artery aneurysm caused restricted damage to the hippocampus unilaterally, and caused memory disturbances.

**Case presentation:**

A 56-year-old, right-handed man, with a formal education history of 16 years and company employees was admitted to our hospital because of a consciousness disturbance. He was diagnosed as having a subarachnoid hemorrhage due to a left posterior cerebral artery dissecting aneurysm, and coil embolization was performed. Subsequently, he had neither motor paresis nor sensory disturbances, but he showed disorientation, and both retrograde and anterograde amnesia. Although immediate recall and remote memory were almost intact, his recent memory was moderately impaired. Both verbal and non-verbal memories were impaired. Brain computed tomography (CT) and magnetic resonance imaging (MRI) revealed a cerebral hematoma in the left temporal lobe involving the hippocampus and parahippocampal gyrus, and single-photon emission computed tomography (SPECT) demonstrated low perfusion areas in the left medial temporal lobe.

**Conclusions:**

We suggest that the memory impairment was caused by local tissue destruction of Papez’s circuit in the dominant hemisphere due to the cerebral hematoma.

## Background

Cognitive disorders, such as memory disturbances, are often observed following a subarachnoid hemorrhage. Basal forebrain amnesia due to aneurysm rupture of the anterior communicating artery was reported more than 50 years ago [1,2]. Anatomically, the basal forebrain is a region consisting of the external and internal septal nuclei, the basal nucleus of Mynert, and the diagonal band of Broca. It is believed that cholinergic neurons that regulate cerebral blood flow by projecting to the cerebral cortex, hippocampus, and amygdala are present in this structure. It has also been reported that damage to the lower branch of the corpus callosum, which is a penetrating branch from the anterior communicating artery, may cause amnestic syndrome with spontaneous confabulation or impaired attention [3-6]. Moreover, memory disturbances are seen after surgery on cerebral aneurysm of the basilar tip. It is believed that the primary cause of this is damage to the hippocampus, hippocampal gyrus, amygdala, fornix, etc., which form the perfusion region of the posterior cerebral artery. This damage may result in a circulatory disorder, or occlusion of the paramedian thalamic artery and thalamogeniculate artery bifurcating from the posterior cerebral artery that sends blood to the diencephalon, or occlusion of the polar artery bifurcating from the posterior communicating artery [7-9]. Aneurysm rupture of the middle cerebral artery, in cases where a hematoma is formed in the cerebral parenchyma, can result in persistent neurological symptoms such as motor paralysis. However, there are hardly any reports of only memory disturbance. Here, we report a case of amnestic syndrome and formation of a hematoma inside the left temporal lobe due to a dissecting aneurysm rupture of the distal portion of posterior cerebral artery.

## Case presentation

A 56-year-old, right-handed man, with a formal education history of 16 years and company employees (office work in an insurance company) was admitted to our hospital because of a consciousness disturbance on 3^rd^ January, 2011. He did not have any history of medication. On that day, he lost consciousness immediately after going to the toilet and his family requested emergency treatment. Subarachnoid hemorrhage due to an aneurysm rupture of the left posterior cerebral artery was diagnosed, and coil embolization was performed on the same day. After interventional surgery, the case was referred to our department for rehabilitation.

### Neurological examination (2^nd^ day after surgery)

The patient was almost completely conscious; however, disorientation with respect to date, place, and age were found. Although neck stiffness was confirmed, no abnormalities were found in the nervous system, and there were no obvious movement disorders. No abnormalities in the sensory system and no ataxia or coordination disturbance was observed. He was cooperative during the examinations. Although aphasia and apraxia were not found, disorientation of time and place originating from anterograde amnesia was confirmed. He was aware of being hospitalized due to illness, and there was no confabulation. Anterograde amnesia was severe, and he had no memory of what he did in relation to the new year and what he ate on the previous day of illness. When responding about the instance where ‘he collapsed in the toilet during the new year’, he repeatedly asked ‘Oh, I do not know at all. Is that true? Oh, well, where and when did I collapse?’ and he was not able to remember even the most recent conversation. In addition, his memory for the approximate 1-month period prior to illness was also vague. On the other hand, he could correctly answer all questions related to his educational and career background, names of his children, etc. Table 1 shows the results of neuropsychological tests. He stopped attempting to answer some questions, as he could not remember them. Although performance was somewhat reduced in the intelligence test, overall cognitive function was intact, and immediate memory was also maintained. Decline in performance was found in all memory related tests, and the effect of verbal and speech learning was also poor. Although mild frontal lobe function disorder was found, depression and anxiety were absent.

**Table 1:** Results of neuropsychological tests

	**1 week**	**4 weeks**	**10 weeks**	**Mean (SD) of healthy**
Mini-mental state examination (/30)	21	27	28	29.0 (1.5)
Digit span				
forward	8	8	8	
backward	4	5	4	
Raven’s progressive matrices (/36)	31	36	34	29.4 (3.7)
Frontal assessment battery (/18)	14	17	14	14.3 (1.5)
Wechsler adult intelligence scale 3rd edition				
Verbal IQ	88	99	90	100 (15)
Performance IQ	70	79	90	100 (15)
Trail making test				
A	218	130	123	109.3 (35.6)
B	376	150	220	150.2 (51.3)
Behavioural assessment of dysexecutive syndrome	53	88	102	
Age matched score < classification>	impaired	under average	average	
Auditory verbal learning test (/15)				
Immediate recall				
first	3	3	4	4.8 (1.4)
second	4	5	7	7.2 (1.9)
third	5	6	6	8.9 (2.2)
forth	5	5	9	9.8 (2.3)
fifth	4	5	6	10.6 (2.1)
Recognition	13	15	14	13.7 (1.9)
Delayed recall	0	0	3	8.8 (3.0)
Wechsler memory scale-revised				
Verbal memory	52	73	79	100 (15)
Visual memory	under 50	52	70	100 (15)
General memory	under 50	61	72	100 (15)
Concentration/attention	98	106	117	100 (15)
Delayed recall	under 50	under 50	under 50	100 (15)
Rivermead behabioural memory test				Cut-off score
Screening score(/12)	0	0	0	7/8
Profile score(/24)	2	5	5	16/17

### Neuroradiological examination

**Figure 1:**
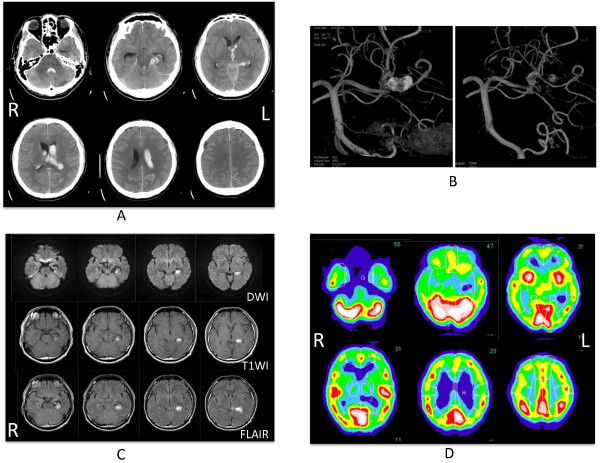
**Neuroradiological examination. (A)** A hematoma was observed in the left hippocampus, extending to the parahippocampal gyrus in a brain CT scan on admission. **(B)** Cerebral angiography before (left) and after (right) coil embolization. An irregular dissecting aneurysm was found in the distal portion of the left posterior cerebral artery. Dissection had occurred in the main trunk of the posterior cerebral artery, and it was formed due to a portion that extended saccular and a portion that extended in fusiform, where the portion that extended in saccular was embolized, and the main trunk was preserved. **(C)** A hematoma was found in the left hippocampus extending to the parahippocampal gyrus in MRI scan on day 10 after the onset. **(D)** A decline in cerebral blood flow in the left medial temporal lobe was found in SPECT images on day 14 after the onset.

In addition to subarachnoid hemorrhage, a hematoma was observed in the left hippocampus, extending to the parahippocampal gyrus in a brain CT scan (Figure 1a). In cerebral angiography, an irregular dissecting aneurysm was found in the distal portion of the left posterior cerebral artery. Dissection had occurred in the main trunk of the posterior cerebral artery, and it was formed due to a portion that extended saccular and a portion that extended in fusiform, where the portion that extended in saccular was embolized, and the main trunk was preserved (Figure 1b). A hematoma was found in the left hippocampus extending to the parahippocampal gyrus on then MRI scan on day 10 after the onset of the symptoms (Figure 1c). In SPECT images on day 14 after the onset of symptoms, around the same area, a decline in cerebral blood flow in the left medial temporal lobe was found (Figure 1d).

### Clinical course

The clinical course after the endovascular surgery was good, and symptomatic cerebral vasospasm or hydrocephalus was not observed. Although the patient began to perform daily living activities independently, due to the memory disorder, he had difficulties in schedule management and medication compliance. As overall cognitive function was good, we instructed him to keep a note on the desk as an external support strategy, and to note down details of conversation and his daily schedule, to-do list, etc. Although improvements were seen during the course of his treatment, memory disturbance remained. However, as it became possible to independently manage his life by learning the use of note-taking, he was discharged after 10 weeks.

## Discussion

Amnestic syndromes are conditions in which memory functions are disproportionately impaired compared to other cognitive functions in otherwise alert patients [8,9]. In the 1950s, research conducted on memory impairment due to hippocampal lesions was mainly after resection in patients with serious mental disorders or temporary lobe epilepsy. Scoville et al. [10] studied memory impairment after resection of the mesial temporal lobe, and reported that memory impairment is found only when there is invasion on both sides of the hippocampus. Moreover, Penfield [11] suggested that occurrence of memory disturbances on one side is due to a cryptic lesion on the opposite side, and he emphasized that lesions on either side can cause memory impairment. Thereafter, it was reported that relatively mild memory disturbances may be observed due to unilateral lesion, and that mainly verbal memory is impaired due to a left-sided lesion, while non-verbal memory is impaired due to a right-sided lesion; however, temporary memory loss is seen in most cases of unilateral lesion [12,13]. In recent years, cases of not only verbal memory impairment, but also visual memory impairment and prolonged amnesia due to unilateral lesion have also been reported. In the present case of unilateral lesion of the left side, verbal memory, as well as non-verbal memory, was impaired [14]. This supports the hypothesis that there may be individual variation in verbal and non-verbal lateralization related to memory.

With regard to neural circuits related to memory, the Papez circuit that consists of the hippocampus, a group of anterior thalamic nuclei, the medial nucleus of the mammillary body, and the hippocampal gyrus, and the Yakovlev circuit that consists of the amygdala, medial nucleus of thalamus, and interior surface of the frontal cortex have been implicated. Although these circuits are independent, they have a strong mutual relationship [15]. The damage limited to bilateral hippocampus causes anterograde amnesia, but it does not necessarily cause retrograde amnesia [16]. Severe retrograde amnesia was caused by damage to both of parahippocampal gyri with hippocampus. A widely distributed network of regions underlies the retrieval of past memories, and that the extent of lateral temporal damage appears to be critical to the emergence of severe remote memory impairment was discussed [17]. Since retrograde amnesia was mild and remote memory was maintained, we supposed that the pathological lesion of our case was limited to the unilateral hippocampus and parahippocampal gyri.

In stroke patients, cerebral infarction is the most frequent cause of memory impairment [7,9,14], and in many cases it occurs as the result of a thalamic or hippocampal lesion. Moreover, with regard to the site of cerebral hemorrhage, pathology of the caudate nucleus, the anterior region of the thalamus, or the anterior part of the splenium of the corpus callosum, have also been reported. On the other hand, memory impairment due to subarachnoid hemorrhage may be caused by several factors such as diffuse brain damage due to the subarachnoid hemorrhage itself, intracerebral hematoma, an invasive surgical procedure, or the effect of cerebral vasospasm and hydrocephalus.

As postoperative complications following cerebral aneurysm of the posterior circulation system, damage to the hippocampus, hippocampal gyrus, amygdala, fornix, etc., which forms the perfusion region of the posterior cerebral artery, causes circulatory disorder due to occlusion of the initial portion. Additionally, the paramedian thalamic artery from the posterior cerebral artery P1 section, the thalamogeniculate artery from the P2 section, or the polar artery from the posterior communicating artery sends blood to the diencephalon after branching, and particularly when approached infratemporally, there is risk that penetrating branches or the posterior cerebral artery will be obstructed [18,19]. In the present case, coil embolization was performed. However occlusion of the posterior cerebral artery or cerebral embolism of its penetrating branches was not found in the postoperative MRI. Moreover, during the treatment course, symptomatic cerebral vasospasm or hydrocephalus was not observed, and overall cognitive function such as immediate recall and intelligence remained consistent. In addition, in SPECT images, diffuse brain damage was not observed, and functional lesions were restricted to the left medial temporal lobe where the hematoma was present. Rather than storage of memory or memory retrieval, the hippocampus is an important site for encoding the memory, and it is involved in fixation of the memory in a specific time in the past [20]. Therefore, when the hippocampus is damaged, retrograde amnesia is not so severe (in most of the cases it is restricted to a few years before the onset of symptoms), and remote memory remains intact. Accordingly, in the present case, retrograde amnesia was restricted to a few weeks before the onset of symptoms, and remote memory was intact. Moreover, spontaneous confabulation or attention disorders, often seen in basal forebrain amnesia [6], were absent.

## Conclusion

This was a very rare case where rupture of a posterior cerebral artery aneurysm caused restricted damage to the hippocampus unilaterally, and caused memory disturbances. We believe that this is an important case for confirming the role of the hippocampus in memory.

### Patient consent

Written informed consent was obtained from the patient for publication of this case report and any accompanying images. A copy of the written consent is available for review by the Editor-in-Chief of this journal.

## Competing interests

The authors declare that they have no competing of interests.

## Authors’ contributions

SM conceived the study, participated in its design and coordination, and helped to draft the manuscript. AO searched the literature and helped to draft the manuscript. FY and HS critically revised the manuscript for important intellectual content. IO, RK, TY, NU and SI participated in the design of the study, helped to draft the manuscript, and performed the statistical analysis. All authors read and approved the final manuscript.

## Pre-publication history

The pre-publication history for this paper can be accessed here:

http://www.biomedcentral.com/1471-2377/14/44/prepub
